# Intersecting dilated convex polyhedra method for modeling complex particles in discrete element method

**DOI:** 10.1002/nag.2299

**Published:** 2014-05-15

**Authors:** Ben Nye, Anton V Kulchitsky, Jerome B Johnson

**Affiliations:** 1 Institute of Northern Engineering, University of Alaska FairbanksFairbanks, AK 755910, U.S.A.

**Keywords:** discrete element method, granular materials, shape of elements, polyhedral particles, concave particles, dilated particles

## Abstract

This paper describes a new method for representing concave polyhedral particles in a discrete element method as unions of convex dilated polyhedra. This method offers an efficient way to simulate systems with a large number of (generally concave) polyhedral particles. The method also allows spheres, capsules, and dilated triangles to be combined with polyhedra using the same approach. The computational efficiency of the method is tested in two different simulation setups using different efficiency metrics for seven particle types: spheres, clusters of three spheres, clusters of four spheres, tetrahedra, cubes, unions of two octahedra (concave), and a model of a computer tomography scan of a lunar simulant GRC-3 particle. It is shown that the computational efficiency of the simulations degrades much slower than the increase in complexity of the particles in the system. The efficiency of the method is based on the time coherence of the system, and an efficient and robust distance computation method between polyhedra as particles never intersect for dilated particles.

## 1. INTRODUCTION

### 1.1 Background

Most 3D discrete element method (DEM) models use spherical particles because of their efficiency and simplicity. However, this approach leads to many oversimplifications that can produce unrealistic results. For example, representing solid bodies as unions or clusters of spheres will always have cavities regardless of the diameter distribution of the spheres used to represent the solid. As a result, porosity is unavoidable in modeling solid materials, which can affect the ability to accurately simulate microscale mechanical deformation processes. Another difficulty occurs when trying to represent particle interlocking and dilation that occurs during the deformation and failure of granular materials 1. The degree of particle interlocking and dilation that occurs during granular material deformation and failure is a strong function of particle angularity. Spherical particles are not well suited to represent angular particles as their ability to interlock with neighboring particles is limited. Efforts to construct a single angular particle would require a prohibitively large number of spheres where a simple polyhedron would suffice.

One of the earliest DEM codes to simulate 3D convex polyhedral particles was developed by Ghaboussi and Barbosa 2. Their method was modified to include more efficient distance calculation methods, such as the shortest link method 3, and more sophisticated contact mechanics, such as the Hertzian model for normal forces and an iterative model for tangential forces 4, as well as taking into account types of features of polyhedra that are in contact 5.

Dealing with intersections between particles is one of the significant complications in modeling particles as polyhedra. Many algorithms for calculating distance (e.g., GJK 6) either do not work in the case of overlapping polyhedral particles or work inefficiently. An efficient approach to avoid complications associated with particle intersection is to introduce a dilation radius, a small rounding boundary around the polyhedron that is considered to be a part of the particle 7,8. The dilation is achieved by stepping a small distance away from the surface of the original polyhedron in all outside directions, forming an envelope around the polyhedral frame. This is also known as a safety margin 9, or as particles with rounded edges 10,11. The dilation radius for DEM particles should be large enough to guarantee that the overlap between two particles never exceeds the dilation radius. This ensures that there are never any pairs of intersecting polyhedral frames in the system. For normal stiff particles, the minimum safety dilation is negligible compared to the size of the contacting particles. This is adequate for contact mechanics assumptions to remain correct for the system 12. The usage of a dilation radius greatly simplifies handling contacts and, as follows from this paper, logically unites both polyhedral and spherical representations of particles. There are no physical restrictions on a maximum size of the dilation radius.

Most DEM codes use convex polyhedra to represent particles because there are no adequate methods to deal efficiently with multiple points of contacts between concave polyhedra, and a pair of convex shapes always have at most one contact. In 8, polyhedra particles are represented as a union of independent spheres, cylinders, and triangular faces that establish independent contacts with each other. This allows concave shapes to be simulated but makes it difficult to accurately calculate the particle's mechanical properties, such as center of mass and inertia tensor. This approach also results in counting the same particle overlap between two particles multiple times, which leads to improper contact mechanics. Although some culling of extraneous contacts is possible, it does not get rid of all multiplied artificial contacts and creates particles with many independent parts that can reduce computational efficiency. For example, to represent a simple cube requires 12 triangular faces, 8 spheres, and 12 cylinders as independent contacting instances in the model. The computational efficiency of simulations is significantly degraded by the complexity of using many independent parts in the polyhedra representation.

### 1.2 Outline

We present a novel method of modeling arbitrary DEM polyhedral particles by a union of convex dilated polyhedra with triangular faces or the ‘Intersecting Dilated Convex Polyhedra’ (IDCP) method. This method is inspired by a simplified version of the constructive solid geometry method for representing complex bodies 13 and extends the method reported in 14. The IDCP method allows a polyhedron of any complexity to be represented in a simple and computationally efficient way.

In this paper, we demonstrate the implementation of the IDCP method in the COUPi (Controlled Objects Unbound Particles interaction) DEM model developed at the University of Alaska Fairbanks. Several different metrics of computational efficiency are introduced, and two different types of computations are performed: gravitational deposition and particles in a rotating drum, for seven different particle shapes from simple spheres to complex concave polyhedra representing realistic particles. Performance of the model in these tests is analyzed for different particle shapes.

## 2. PARTICLE REPRESENTATION

**Figure 1:**
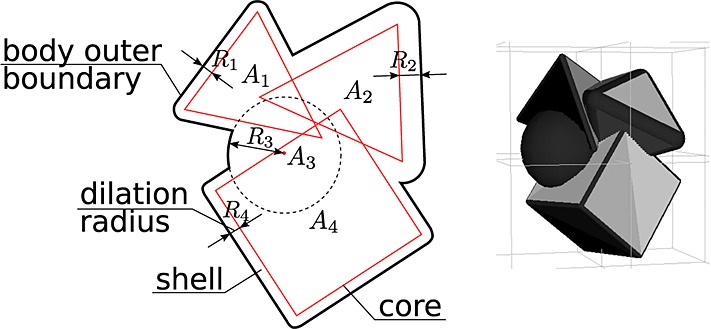
Complex body representation by a union of convex polyhedra subshape atoms. A particle body is represented as a union of four atoms *A*_1_,*A*_2_,*A*_3_, and *A*_4_ with different dilation radii *R*_1_,*R*_2_,*R*_3_, and *R*_4_. The cores of atoms are the actual frames of polyhedra subshapes (red). The core of atom *A*_3_ is an isolated vertex that makes the atom a sphere of radius *R*_3_. A *shell* is a part of the atom that is formed by a dilation radius. The outer boundary of all shells form an outer boundary of the body.

The basic concept of the IDCP method is that every physical rigid polyhedral particle, called a *body*, can be represented as a union of convex dilated shapes called *atoms* that are defined as indivisible geometrical polyhedral subshapes (e.g., cubes or a tetrahedra) as shown in Figure 1. An example of a trivial atom is a dilated point, which is a sphere. The atoms that compose a body may overlap in any way, and the atoms may be arbitrarily complex polyhedra so long as they remain convex. This representation allows us to implement the following:

- The ability to simply calculate physical properties, such as volume and the moment of inertia tensor, of particles. Because checking if a point is in a body is simple (a point is inside of a union of convex polyhedra if and only if it is inside of at least one element), numerical calculation of integrals over particle volume is straightforward.
- The ability to use a variety of well studied methods for computing distances between particles because contacts are calculated between atoms that have polyhedral frames that are always convex and nonintersecting. The distance algorithm implemented in the IDCP method offers near-constant performance regardless of atom complexity and leverages time coherence to offer very good performance 15.
- The IDCP method is general enough to include spheres (as dilated vertices), capsules (as dilated edges), and dilated triangular faces as individual parts of bodies or individual particles. Moreover, the overhead to consider spheres as isolated convex polyhedra with one vertex is minimal and is as efficient as conventional sphere based codes. Bodies can be composed of a mixture of spheres and polyhedra.

Concave bodies can be constructed from several atoms—because any concave polyhedron can be decomposed into some number of convex polyhedra, this method does not restrict the types of particles that can be simulated. Each atom is composed of some number of vertices, edges, and triangular faces which are collectively called features.

**Figure 2:**
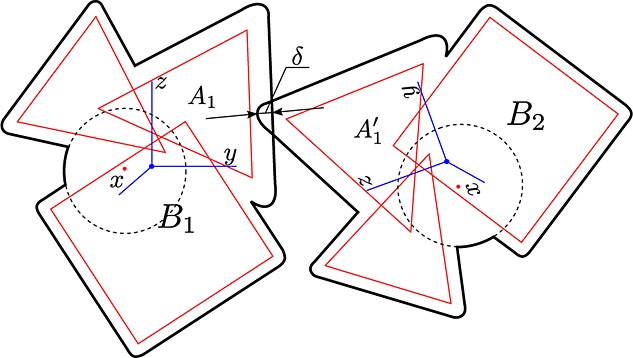
An example how two bodies *B*_1_ and *B*_2_ contact each other by their atoms *A*_1_ ∈ *B*_1_ and 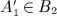. The overlap of the two particles is *δ*, which is determined by the magnitude of normal forces acting at the contact and the material stiffness (Section 3.3). Each atom is assigned material properties separately. Contact properties are defined by a pair of interacting materials. All atoms within a body are defined using body local coordinates *x*,*y*,*z* (blue) with a center in the body center of mass and axis parallel to principle axis of inertia. The global coordinates of every body point are defined by the location of the center of mass and the body orientation.

Each atom has an associated dilation radius, *R*. All points within *R* of the surface of the polyhedron are also in the atom. Two atoms from different bodies are in contact with each other if their dilated envelopes overlap as shown in Figure 2. This means that atoms are in contact only if the minimum distance between the undilated atoms is less than the sum of the two atoms’ dilation radii. Finding the minimum distance between two noninsersecting convex polyhedra is well studied and can be performed efficiently 15–17.

## 3. MODEL DESCRIPTION

### 3.1. Contact detection

Finding contacts between bodies is performed in two stages: a fast broad phase stage and a slower exact narrow stage that is used for particles that are potentially in contact. Each atom of a body is surrounded by a bounding sphere, and on each broad phase contact detection step, any overlapping bounding spheres of atoms from different bodies are flagged as indicating a potential contact. The model uses a hybrid contact detection scheme based on space partitioning in columns and performing a sweep and prune algorithm on each column 18. These potential contacts are accumulated and passed to the distance calculation algorithm that verifies if a contact occured or not and finds the overlap distance for actual contacts. Generating the potential contact list is computationally expensive and is not performed every time step. Instead, the bounding spheres are expanded to encompass the potential movement of that atom between contact detection steps so the contact list contains all potential contacts that could occur between the current time step and the next contact detection time step. The model automatically defines the moment when the next contact detection operation is necessary by tracking the positions of the centers of bounding spheres.

### 3.2. Distance calculation

Once a list of pairs of potentially contacting atoms is generated, we must compute the distances between each pair to determine if the dilated atoms overlap. For any two convex polyhedral atoms, there is a point on the undilated surface of each atom that is closest to the other atom. The feature of each atom that contains that closest point is called the closest feature. Finding the distance between any pair of features can be computed using known geometric methods 19, so we can easily find the distance between two atoms if we can determine the closest feature pair.

Because distance calculations are performed every time step and atoms move very little between each time step, the closest feature pair for two contacting atoms is usually the same as on the previous time step, or at least in the same neightborhood on the surface of the atom. We use an iterative algorithm described by Lin 15 that starts from an initial guess for the closest feature pair and iteratively checks each of the candidate closest features to see if any of their adjacent features are closer to the feature on the other atom, walking to closer adjacent features when possible to converge to the solution. Once the closest feature pair for a pair of atoms is found, the value is stored in a global hash table and used as the initial guess for the computation on the next time step.

**Figure 3:**
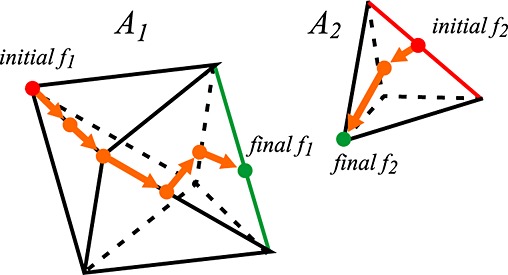
A potential walk along the surfaces of two atoms to find the closest feature pair.

Given a feature *f*_1_ on atom *A*_1_, and a feature *f*_2_ on atom *A*_2_, we first check to see if any of the adjacent features of *f*_1_ are closer to *f*_2_. If any of them are, we replace *f*_1_ with that feature and repeat the test. This process is effectively traversing the surface of *A*_1_ until we have minimized the distance to *f*_2_. When we have found the closest *f*_1_, we repeat the same process, this time checking *f*_2_ for adjacent features closer to *f*_1_ and walking along the surface of *A*_2_. After updating the potential closest feature of one atom, we must check that the potential closest feature on the other atom is still the closest. If not, we walk the surface again to find a new closer feature. Each time we repeat this process, the distance between our two potential features decreases until we reach the global minimum, as illustrated in Figure 3.

For any pair of adjacent features, there is a separating plane that divides space such that all points on one side of the plane are closer to the first feature and all points on the other side of the plane are closer to the second feature. To check if an adjacent feature of *f*_1_ is closer to *f*_2_ or not, we compute the separating plane between *f*_1_ and the adjacent feature and then check which side of the plane the point from *f*_2_ is on.

**Figure 4:**
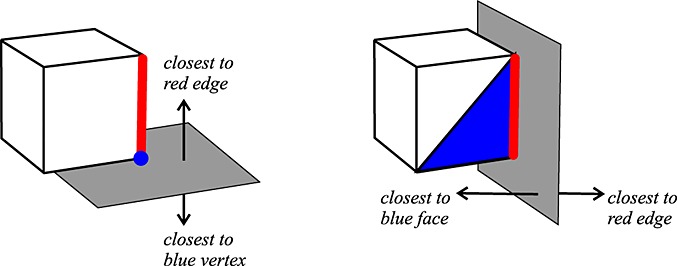
Separating planes for feature pairs. First, an edge and a vertex, then an edge and a plane. The planes partition space depending on which of the features is closer to points on each side.

Vertices and faces are not considered to be adjacent, so we only ever need to find separating planes for vertex–edge pairs and edge–face pairs. For an vertex–edge pair, the plane is perpendicular to the edge and contains the vertex. For an edge-face pair, the plane is perpendicular to the edge and the face normal and contains the edge, as shown in Figure 4.

### 3.3. Contact mechanics

Interaction between contacting bodies causes forces on every contacts. In the COUPi DEM model and in this work, contact forces are represented by

- normal elastic forces
- adhesive forces
- tangential forces, and
- normal and tangential damping forces.

The forces are computed using a modified Hertz–Mindlin model.

**Figure 5:**
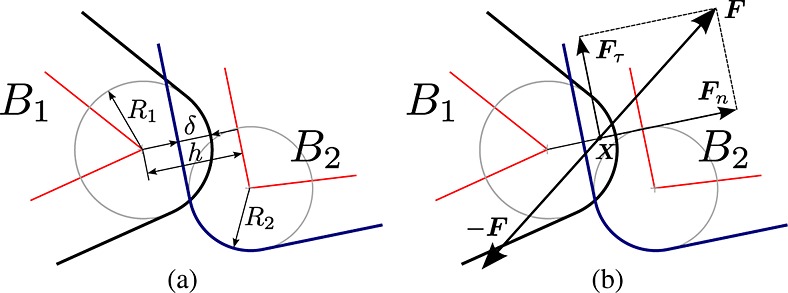
Example of two bodies *B*_1_ and *B*_2_ interacting. (a) *B*_1_ outer boundary (black) intersects *B*_2_ outer boundary (blue) with overlap *δ* = *R*_1_ + *R*_2_ − *h*, where *R*_1_ is a dilation radius of interacting atom of body *B*_1_ and *R*_2_ is a dilation radius of interacting atom of body *B*_2_. (b) If *X* is a middle point of segment *δ*, then force *F* acts on body *B*_2_ and applied at *X*. Opposite force acts on *B*_1_.

*The elastic normal component* of the contact force is calculated using a modified Hertzian model 12. The contact force depends on the overlap between particles (Figure 5). The overlap between atom *A*_1_ and atom *A*_2_ is defined as 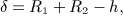
1 where *h* is the distance between the undilated cores of the atoms. *R*_1_ and *R*_2_ are dilation radii of the two atoms. As mentioned earlier, dilation radii are chosen such that *h* > 0. Following 12, the elastic component of the contact force can be found as follows: 
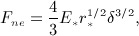
2 where the effective Young's modulus *E*_ * _ and effective interaction radius *r*_ * _ are defined as follows: 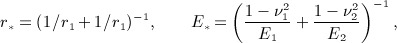
3 where *r*_1_ and *r*_2_ are the radii of curvature at the contact point, *ν*_1_ and *ν*_2_ are Poisson's ratios, and *E*_1_ and *E*_2_ are Young's modulus of two atoms *A*_1_ and *A*_2_.

Although the contact between two polyhedra depends on the feature types they are contacting with 20 (i.e., the normal force formula is different for vertex–vertex and edge–edge contacts), the simplified contact model is used in this work 21,8. We assign an artificial curvature radius to each whole atom equal to a sphere of equal volume. This is justified by the fact that there are no truly flat or smooth surfaces in real systems, so a contact is defined on a rough surface, and we use some average value for the interaction curvature radius. In addition, unless there is a strong confining pressure or very low interparticle friction ratio, even in pure geometrical polyhedral granular materials, the frequency of face–face contacts is small, and they seldom need to be accounted for as most of the contacts are vertex–face, vertex–edge, and edge–edge contacts. For such contacts, curvature affects the effective stiffness on the contact, and stiffness plays a minor role compared to geometry. Our tests further address only these types of systems.

*Adhesive normal component* of the contact force is calculated using Johnson, Kendall, and Roberts 22,12 model with linearization proposed in 23,24.

*Normal damping component F*_*ni*_ is used from the extended Hertz theory 25, 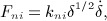
4 where the constant damping coefficient *k*_*ni*_ nonlinearly depends on the coefficient of restitution 26, 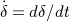.

*Tangent force* 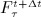 value at the next time step *t* + Δ*t* is calculated incrementally from the force at the previous time step similar to 4. The computation formulas can be expressed as follows. 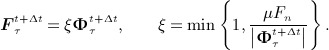
5

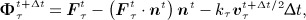
6 where *μ* is the Coloumb coefficient of friction between two particles, *k*_*τ*_ is the tangential contact stiffness, ***n***^*t*^ is the normal vector to the contact plane, and 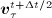 is the relative velocity of the particle at the point of contact.

### 3.4. Dynamics

Knowing all the forces and torques acting on particles, their velocities and angular velocities are calculated from the numerical approximation of the Newton and Eular equations 27.

### 3.5. Orientation and position

Rigid body motion is described by the change in position of a particle's center of mass and rotation around it. The new position of the center of mass of each body in the system is defined by the integration of the equation of motion. Orientation of the body is defined by the integration of rotation equation 27.

## 4. TESTS

### 4.1. Particles

**Figure 6:**
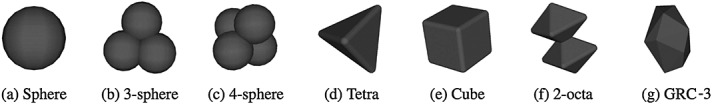
Experimental particle types from fewest features to most features.

We ran tests with seven different particle types of different complexities, shown in Figure 6, in order of increasing number of features.

The estimated running time of the distance calculation is 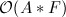, where *F* is the number of features the algorithm checks as potential closest features and *A* is the average number of adjacent features. When the initial guess for the closest feature pair is correct, only those two features need to be checked. Experimentally, *F* is very close to 2 regardless of particle type, usually about 2.1 for the polyhedral particles shown in Figure 6. As a result, the scalability of the distance calculation depends primarily on *A*. Faces and edges always have the same number of adjacent features, and vertices can be adjacent to any number of edges. While more complicated particles do tend to have vertices of higher degree, *A* increases slowly relative to the complexity of the particle. This means the distance algorithm offers very good near-constant expected run time for different particle types.

**Figure 7:**
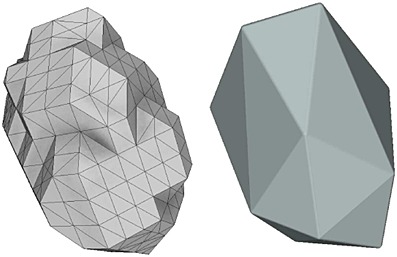
GRC-3 lunar simulant particle 3D computer tomography scan (left) and simplified model used in the tests (right).

Spheres were expected to perform the best in all circumstances because the distance algorithm does not need to check any adjacent features for atoms composed of a single vertex, and only needs to compute the distance between the vertices that are the centers of the two spheres. Additionally, spheres perfectly fit their bounding spheres, which makes finding potential contacts for spherical particles very accurate. The three-sphere and four-sphere particles also benefit from a trivial distance atom–atom distance calculation but have more contacts per particle. The tetra particle was chosen as the simplest polyhedral particle. Because we limit ourselves to triangular faces, each side of the cube particle is composed of two triangular faces and one diagonal edge and the particle in total requires over 2.7 times as many features as the tetra. The two-octa particle was chosen to be a representative more complex, multi-atom concave body. The GRC-3 particle was modeled after a computer tomography scan of a lunar regolith simulant GRC-3 particle as shown in Figure 7. Table 1 shows the composition details for each particle type.

**Table I:** The number of features each particle type is composed of.

Particle type	Vertices	Edges	Faces	Total	Volume	Adjacent features
Sphere	1	0	0	1	1.00	0
Three-sphere	3	0	0	3	0.48	0
Four-sphere	4	0	0	4	0.62	0
Tetra	4	6	4	14	0.35	3.43
Cube	8	18	12	38	0.46	3.69
Two-octa	12	24	16	52	0.28	3.79
GRC-3	13	33	22	68	0.33	3.88

Note that each side of the cube is composed of two triangular faces and a diagonal edge. Volume is measured as a percentage of bounding sphere. Last columns indicates the average number of ajacent features for each polyhedral particle.

### 4.2. Performance metrics

When judging the performance of each particle type, there are several complications that require consideration. The total run time of a trial is highly dependent on the method for generating the list of potential contacts. Because we perform the same distance calculation for every potential contact, generating too many false potential contacts will significantly decrease performance relative to a test that has a more accurate contact list generation. In our implementation, potential contact generation is performed with bounding spheres. This means that our contact list generation is most accurate when particles closely fit their bounding spheres.

The particle type also affects how many contacts need to be computed per time step. Particles that pack more closely or have more average contacts per atom produce much more computationally expensive simulations.

With these concerns in mind, we present three different efficiency metrics:

- *Actual contacts per second*. This measures how the particle type performs while accounting for the unavoidable differences in particle–particle interactions. This metric favors particle types with more accurate contact lists, because any false positives are wasted computation time. We measure this metric in millions contacts processed per 1 s of execution time.
- *Potential contacts per second*. This measures how well the the distance algorithm performs for each particle type. The distance algorithm is one of the main motivations for using this representation and is typically the bottleneck of the computation. This metric favors particle types with less accurate contact lists, because any false positives do not need to have forces computed for that contact. This is also measured in millions of potential contact pairs per 1 s of execution time.
- *Run time*. This metric is very practical from a user's point of view. It strongly favors particles with very accurate contact lists and fewer contacts per body and ignores any issues produced by more complex particle–particle interactions.

Calculation efficiency in DEM simulations depends on many factors, and one of the most important factors is how well the potential contact list is created at the broad-phase contact detection stage. For every pair of atoms in the potential contact list, a distance calculation needs to be performed. To measure how well the potential contact list is created, we record the ratio between the actual number of contacts to the potential number of contacts. This ratio depends on particle type and simulation type and never exceeds 1.

Contacts per body count also influences the performance and depends on the particle type.

Simulations were run as a single threaded process on a PowerMac Laptop with 2.6 GHz Intel Core i7 running OS X 10.8.4.

### 4.3. Physical parameters

The material parameters that were used in the following simulations are shown in Table 2.

**Table II:** Material and contact properties used in the tests. The material stiffness was reduced to increase the time step for the computations.

Material stiffness *G*, GPa	Particles	Drum	Particle–drum
Stiffness, *G*, GPa	0.01	0.07	—
Poisson coefficient, *ν*	0.3	0.31	—
Material density, *ρ*, kg/m ^3^	3000	7861	—
Friction ratio, *μ*	0.7	0.3	0.42
Coefficient of restitution (at 1 m/s), *C*_*R*_	0.5	0.7	0.61
Tangential contact stiffness, *k*_*τ*_	0.7	0.7	0.7

The tests were run under uniform gravitation field of 9.8 m/s ^2^. Particle size was set to 1.7 cm in all the performance tests.

### 4.4. Gravitational deposition test

**Figure 8:**
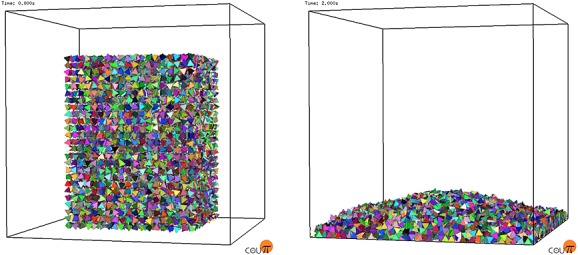
Gravitational deposition initial and final conditions with tetrahedral particles.

We ran simulations for the seven different particle types with 9025 particles from an initial grid and settling into a broad flat pile as depicted in Figure 8. The system dynamics were recomputed every 10  μs of simulation time.

### 4.5. Drum test

**Figure 9:**
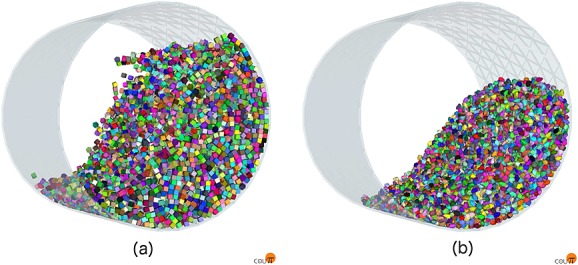
(a) 15,579 cubes and (b) 15,579 GRC-3 model particles rotating in a cylindrical drum (final stage).

We then ran simulations for the seven different particle types with 15,579 particles in a cylindrical drum of radius 0.5 m rotating at *π* radians per second, as shown in Figure 9. The system dynamics were recomputed every 10  μs of simulation time. Statistics were collected once the system had reached a pseudostable state and the particles were tumbling freely. This provided a highly dynamic simulation where all particles were in constant motion relative to each other and many were rotating frequently.

## 5. RESULTS

Across all particles and tests, calculating contact locations and forces dominated the running time. The sphere particle was always the fastest because of the ease of computing contacts. The performance of more complicated particles, including the three-sphere and four-sphere, varied between the two tests due to differences in contact list efficiencies.

### 5.1. Gravitational deposition

In the gravitational deposition tests, the system reached a near-static state after about 0.70 s of the 2.00-s simulation for all particle types. The low particle velocity allowed the potential contact list to be highly accurate for all particle types, regardless of how well they fit their bounding spheres. Because the efficiency of the contact list was almost constant across particle type, as seen in Table 3, differences in contact computation efficiency were due primarily to the performance of the distance algorithm.

**Table III:** Experimental results for different particle types for gravitational deposition.

Particle type	Actual contacts 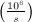	Potential contacts 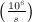	Actual/potential	Contacts/body	Runtime (h)
Sphere	2.19	2.31	0.94	1.79	0.41
Three-sphere	1.80	2.00	0.90	2.63	0.73
Four-sphere	1.67	1.88	0.89	2.81	0.84
Tetra	1.20	1.39	0.86	2.17	0.91
Cube	0.94	1.02	0.92	1.91	1.02
Two-octa	0.92	1.07	0.86	2.43	1.32
GRC-3	0.67	0.82	0.82	2.16	1.61

**Figure 10:**
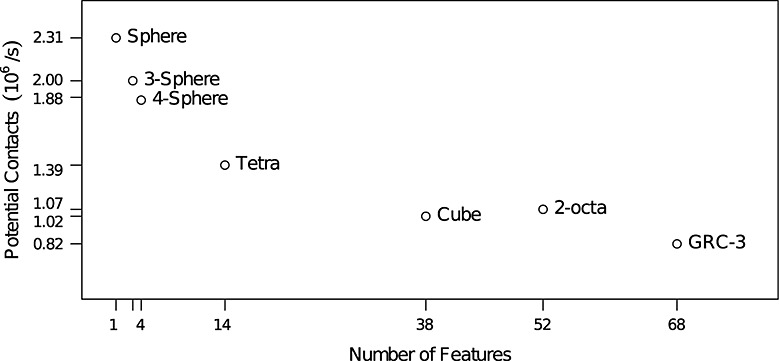
Computational efficiency for different particle types in the gravitational deposition test relative to the number of features in the particle.

The potential contact processing speed is shown in Figure 10. As expected, the three sphere-based particle types offered the highest efficiency because the the distance between two spheres is computed without needing to check any adjacent features. The sphere was 15.5% more efficient than the three-sphere, and 22.9% more efficient than the four-sphere, despite using exactly the same contact calculations and having higher contact list efficiency. This suggests that the increased size of the data structures needed to store the bodies has an adverse effect on efficiency.

**Figure 11:**
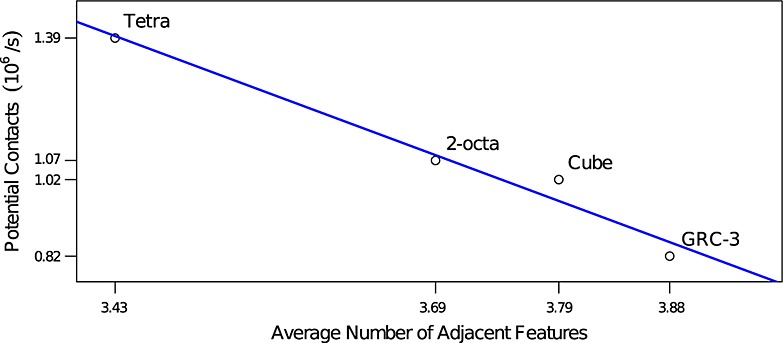
Computational efficiency for different particle types in the gravitational deposition test relative to the average number of adjacent features that need to be checked by the distance algorithm.

The polyhedral particles all had lower efficiency than the sphere-based particles, but scaled well with the number of features. The cube particle was 24.4% more efficient than the GRC-3 particle, but the GRC-3 particle had 78.9% more features. As described in Section 4.1, the average number of adjacent features, *A*, increases slowly as the number of features in the particle increases, going from 3.43 for the tetra particle to 3.88 for the GRC-3 particle. Due to particles being static for most of the simulation time, the number of features checked by the distance algorithm, *F*, is almost always 1. The relationship between *A* and potential contact processing speed is shown in Figure 11 and is linear as claimed in Section 4.1.

### 5.2. Drum

In the rotating drum tests, contact list efficiency varied significantly between different particle types, from 0.84 for the sphere particle to 0.56 for the four-sphere particle. All results are shown in Table 4. The contact list efficiency and the number of contacts per particle played a large role in total run time.

**Table IV:** Experimental results for different particle types in a rotating drum.

Particle type	Actual contacts 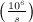	Potential contacts 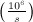	Actual/potential	Contacts/body	Runtime (h)
Sphere	1.69	2.00	0.84	1.53	0.59
Three-sphere	0.93	1.55	0.59	1.95	1.36
Four-sphere	0.92	1.62	0.56	2.24	1.58
Tetra	0.89	1.32	0.67	1.83	1.33
Cube	0.85	1.12	0.75	1.56	1.20
Two-octa	0.61	1.03	0.59	1.87	1.98
GRC-3	0.57	0.86	0.66	1.87	2.19

Because contacts are computed per-atom, bodies composed of several atoms suffered the most degredation in contact list efficiency between the gravitational deposition test and the rotating drum test. Inflating the bounding spheres around each atom allowed for multiple false contacts to be generated if a neighboring body was close enough to intersect several of the bounding spheres around the atoms.

**Figure 12:**
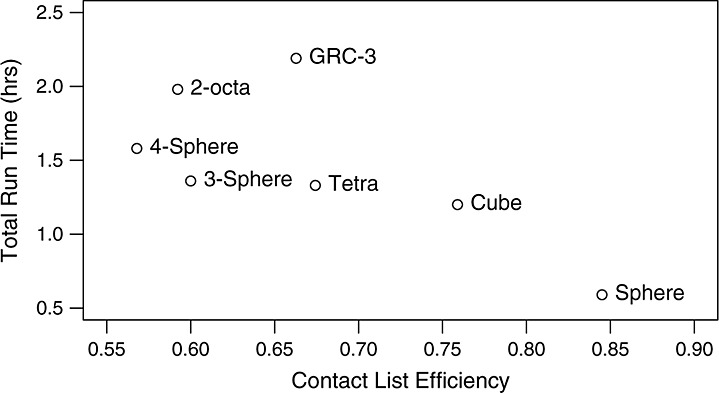
Total run time for different particle types in the rotating drum test relative to the contact list efficiency. Correlation coefficient is − 0.70.

While spheres ran the fastest at 0.59 h, both cubes and tetras ran faster than the more complicated sphere-based particles. Overall, contact list efficiency correlated strongly ( − 0.70) with total run time as shown in Figure 12.

The results of the dynamic case show that the particles produced by the IDCP method are similar in computational complexity, even when the difference in physical complexity is large. The contact algorithm employed here was better suited for cubes than for the much simpler 3-spheres, and this bias dominated the difference in physical complexity. The IDCP method provides very good scaling with particle physical complexity, especially since the bounding volume used to generate the potential contact list can be adjusted to closely approximate the desired particle shapes.

## 6. CONCLUSION

The IDCP method allows for an efficient implementation of complex particles as the union of regular polyhedral atoms. Dilation of the atoms produces a natural representation of spheres as well, allowing for bodies composed of both spheres and polyhedra. Concave bodies can be created as the union of several convex polyhedra.

By restricting atoms to convex polyhedra, and contact mechanics that prevent the undilated polyhedral frames of contacting atoms from ever intersecting, computing the distance between two atoms can be performed with fast algorithms that scale very well with increasing particle complexity even in highly dynamic tests such as rotating drum test.
